# Quality of T-cell responses versus reduction in viral load: results from an exploratory phase II clinical study of Vacc-4x, a therapeutic HIV vaccine

**DOI:** 10.1186/1742-4690-9-S2-O66

**Published:** 2012-09-13

**Authors:** K Ellefsen-Lavoie, J Rockstroh, R Pollard, G Pantaleo, D Podzamczer, D Asmuth, J van Lunzen, K Arastéh, D Schürmann, B Peters, B Clotet, D Hardy, A Lazzarin, J Gatell, MA Sommerfelt, I Baksaas, V Wendel-Hansen, B Sørensen

**Affiliations:** 1 University of Lausanne, Lausanne, Switzerland; 2 University of Bonn, Bonn, Germany; 3 University of California at Davis, Davis, CA, USA; 4 Universitari de Bellvitge, Barcelona, Spain; 5 Universitatsklinikum Hamburg-Eppendorf, Hamburg, Germany; 6 EPIMED, Berlin, Germany; 7 Charité Virchow-Klinikum, Berlin, Germany; 8 Guy's and St. Thomas' Hospital, London, UK; 9 Hospital Germans Trias i Pujol, Badalona, Spain; 10 Cedars-Sinai Medical Center, Los Angeles, CA, USA; 11 Università Vita-Salute San Raffaele, Milan, Italy; 12 Hospital Clinic i Provincial, Barcelona, Spain; 13 Bionor Pharma ASA, Oslo, Norway; 14 Mericon, Skien, Norway; 15 Bionor Pharma, Oslo, Norway

## Background

Immunization with Vacc-4x, a peptide-based therapeutic vaccine for HIV-1, has shown a statistically significant reduction in viral load set point compared to placebo during treatment interruption in an exploratory phase II clinical study enrolling 135 subjects (NCT00659789). This vaccine aims to induce sustained cell-mediated immune responses to conserved domains on HIV p24.

## Methods

After 6 immunizations on ART over 28 weeks, treatment was interrupted for up to 24 weeks (Vacc-4x n=88; placebo n=38). Immunological analyses (ELISPOT, proliferation, intracellular cytokine staining (ICS)) to HIV p24 were carried out at central laboratories. The HLA class I profile (Vacc-4x n=73, placebo n=32) was also determined.

## Results

For subjects that remained off ART until week 52 (Vacc-4x n=56, placebo n=25), there was a log 0.44 reduction in viral load set point between the Vacc-4x and placebo groups (p=0.0397). There was a similar distribution of HLA class I alleles in the two treatment arms, with the exception of the B35 allele (27% of Vacc-4x subjects versus 8% placebo subjects). The viral load of ELISPOT positive Vacc-4x subjects was significantly lower than that of placebo subjects (p=0.023). There was no significant difference in T-cell proliferation responses between Vacc-4x and placebo groups, however, the percentage of subjects showing proliferative CD4 and CD8 T-cell responses to Vacc-4x peptides increased over time only for the Vacc-4x group. ICS analysis showed a predominance of CD8-mediated T-cell responses to p24 that were significantly increased from baseline for the Vacc-4x group (p<0.043) but not for the placebo group(p>0.05). There was also a trend towards higher numbers of polyfunctional T-cells in the Vacc-4x group compared to the placebo group (p=0.188).

## Conclusion

These findings suggest Vacc-4x immunization can influence the quality of immune responses to HIV-1 p24 irrespective of HLA status, and contribute to a reduction in viral load.

